# Virtual human as a new diagnostic tool, a proof of concept study in the field of major depressive disorders

**DOI:** 10.1038/srep42656

**Published:** 2017-02-16

**Authors:** Pierre Philip, Jean-Arthur Micoulaud-Franchi, Patricia Sagaspe, Etienne De Sevin, Jérôme Olive, Stéphanie Bioulac, Alain Sauteraud

**Affiliations:** 1 Clinique du Sommeil, Service d’Explorations Fonctionnelles du Système Nerveux, CHU de Bordeaux, Place Amélie Raba-Léon, 33076 Bordeaux, France; 2 Univ. Bordeaux, SANPSY, USR 3413, F-33000 Bordeaux, France; 3 CNRS, SANPSY, USR 3413, F-33000 Bordeaux, France; 4 Pôle Universitaire Psychiatrie Enfants et Adolescents, Centre Hospitalier Charles Perrens, 121, rue de la Béchade, 33076 Bordeaux, France

## Abstract

Embodied Conversational Agents (ECAs) are promising software to communicate with patients but no study has tested them in the diagnostic field of mental disorders. The aim of this study was 1) to test the performance of a diagnostic system for major depressive disorders (MDD), based on the identification by an ECA of specific symptoms (the MDD DSM 5 criteria) in outpatients; 2) to evaluate the acceptability of such an ECA. Patients completed two clinical interviews in a randomized order (ECA versus psychiatrist) and filled in the Acceptability E-scale (AES) to quantify the acceptability of the ECA. 179 outpatients were included in this study (mean age 46.5 ± 12.9 years, 57.5% females). Among the 35 patients diagnosed with MDD by the psychiatrist, 14 (40%) patients exhibited mild, 12 (34.3%) moderate and 9 (25.7%) severe depressive symptoms. Sensitivity increased across the severity level of depressive symptoms and reached 73% for patients with severe depressive symptoms, while specificity remained above 95% for all three severity levels. The acceptability of the ECA evaluated by the AES was very good (25.4). We demonstrate here the validity and acceptability of an ECA to diagnose major depressive disorders. ECAs are promising tools to conduct standardized and well-accepted clinical interviews.

Face-to-face clinical interview is the gold standard to diagnose mental disorders^1^,^2^. This type of interview is important for communicating empathy and elicit patients’ trust in order to collect accurate symptoms essential for a proper diagnosis^1^,^2^. Different procedures have been proposed to diagnose mental disorders. However a general agreement has been reached to consider guidelines of interview specified in the DSM-5 through a list of criteria to be checked as the most reliable and validated procedure to diagnose a mental disorder^3^. Apart from diagnosis, severity of the disease can be evaluated through questionnaires that have been used to classify patients with mild, moderate or severe mental disorders^2^,^4^.

Several computerized interviews based on questionnaires have been used to evaluate patients with mental disorder^5^,^6^,^7^. Moreover, software programs have been used to deliver computer-aided psychotherapy (CP)^8^,^9^. Such CP have been particularly used to treat patients with Major Depressive Disorder (MDD). For example, The *Beating the blues* CP has been validated by two randomized controlled trials (RCT)^10^,^11^ and has been recommended for use in the NHS by the National Institute for Health and Clinical Excellence (NICE). However, none of these programs have been developed as a face-to-face interview to make a diagnosis of mental disorder based on the DSM-5 criteria^3^. Among software programs, specific virtual agent software has been designed to improve the empathic relation with patients and the acceptability of such solutions. For example, software-driven virtual humans (i.e. embodied conversational agents or ECAs) are able to conduct a clinical interview as a real physician would^5^,^12^,^13^. To do so, ECAs combine verbal, facial and gestural expressions in order to conduct a face-to-face interview^12^,^13^. They thus attract and maintain the attention of patients by making the interaction more emotionally expressive, socially adapted and empathetic^13^,^14^. The SIMSENSEI project by Dr Rizzo and colleagues involved a virtual agent to identify symptoms of depression, anxiety and Post-Traumatic Stress Disorder (PTSD)^15^,^16^. However, these systems have not yet been tested in medical environments to compare the accuracy of a virtual agent versus a trained psychiatrist to diagnose MDD according to the DSM-5 criteria^3^.

MDD is the most frequent chronic mental disorder and it affects 15% of the general population^17^. While it accounts for the main burden of all diseases in terms of “years lost due to disability^18^”, it is still largely under-diagnosed, possibly owing to the duration of clinical face-to-face interviews necessary to diagnose mood disorders. There is therefore a major challenge to develop innovative computerized tools to improve the diagnosis of mood disorders. DSM-5 has listed specific criteria to diagnose properly an MDD, which are now defined as a standard diagnostic procedure^3^. Using ECA software specifically developed to conduct a face-to-face interview based on the DSM-5 criteria to improve diagnosis of MDD in the general population is thus a major challenge for public health.

The aim of this study was to develop a dedicated ECA able to conduct a face-to-face interview, identify specific symptoms and to diagnose MMD on the basis of the DSM-5 criteria^3^. Two hypotheses were tested in the present study. We based the rationale of our work on the principal hypothesis that such an ECA would perform a valid diagnosis of MDD in comparison with a clinical interview conducted by a psychiatrist using the DSM 5 criteria. Because clinical expertise of trained physicians remains the gold standard in psychiatry to validate a diagnosis, we used the clinical interview conducted by a psychiatrist as the reference standard in this study. The secondary hypothesis of our work was that such an ECA would be judged acceptable on an acceptability scale rated by the patients.

## Method

### Participants

Participants were sleep patients referred to our Sleep Clinic in Bordeaux University Hospital recruited from November 2014 to June 2015 in a consecutive sample design. Patients were invited to participate in the study during their routine clinical evaluation for sleep complaints (e.g. insomnia or hypersomnolence complaints). They were not specifically addressed to the Sleep Clinic for depressive symptoms.

After receiving a detailed description of the study, they gave their written informed consent. Informed consent was obtained from all subjects. The study, which was conducted in accordance with the Declaration of Helsinki and French Good Clinical Practices, was approved by the local ethics committee (*Comité de Protection des Personnes – CPP*). The study was classified as a clinical trial by the US National Institutes of Health (ClinicalTrials.gov identifier: NCT02544295, date of registration: September 3, 2015). The present validation article is part of a more general study on the validation of the virtual reality-based diagnosis of neuropsychiatric disorders and sleep/wake disorders (PHENOVIRTPSY).

### Selection criteria

The inclusion criteria were:

- To be aged from 18 to 65 years,

- To be a French native speaker,

- To provide written informed consent.

The exclusion criteria were:

- Insufficient capacity to consent to or understand and answer the self-report questionnaires.

- Mental, visual or auditory deficits interfering with interaction with the ECA.

### Recorded data in the study

Patients completed two clinical interviews a few minutes apart in a randomized order:

- one with the sleep clinic psychiatrist,
- one with the ECA.

At the end of the two interviews, the patients completed the following:

- the Beck Depression Inventory BDI-II to quantify the severity of depressive symptoms^4^,
- the Acceptability E-scale (AES) to quantify acceptability of the ECA^19^.

The BDI-II is a scale originally in English that has been translated and validated in French^20^,^21^. The BDI-II has been used in many previous French studies. The BDI-II consists of 21 items rated by patients according to their experience in the preceding month. The scale ranges from 0 to 63. The severity of depressive symptoms can be determined according to cut-offs suggested by the revised manual of the BDI-II^21^. The following cut-offs of the total score were used: 0 to 13 as non-depressed or minimally depressed, 14 to 19 as mildly depressed, 20 to 28 as moderately depressed, and 29 to 63 as severely depressed.

The AES consists of 6 items rated by the patients on a balanced five-point Likert scale ranging from 1 to 5. The rating was determined according to patients’ experience concerning the evaluation with the ECA. The score was obtained by computing the sum of the scores obtained by items associated with it, from 1 to 5. The scale ranges from 6 to 30. The validated French version of the AES was used^22^.

Gender, age, year of education and antidepressant treatment were collected for each participant.

### Clinical interview conducted by the psychiatrist

The psychiatrist examined the participants and classified those with MDD and those without MDD according to DSM-5 criteria. The psychiatrists and the patients were blind to the diagnosis of the ECA. The diagnostic standard for the present study was expert opinion. However, before starting clinical psychiatric evaluation in this study, the reliability of psychiatrist judgments was checked during a testing session performed by two psychiatrist supervisors on a standardized patient. This session ensured that the psychiatrists evaluated each of the DSM-5 criteria and that a diagnosis of MDD was based on them^3^.

### Clinical interview conducted by a software-driven virtual human

The ECA face-to-face interview can be seen in http://www.sanpsy.univ-bordeauxsegalen.fr/Papers/Additional_Material.html. Each response of the patients to each question of the interview was encoded and a decisional algorithm tree was used to establish a binary classifier system and to classify participants with MDD and those without MDD according to DSM-5 criteria.

**Figure 1:**
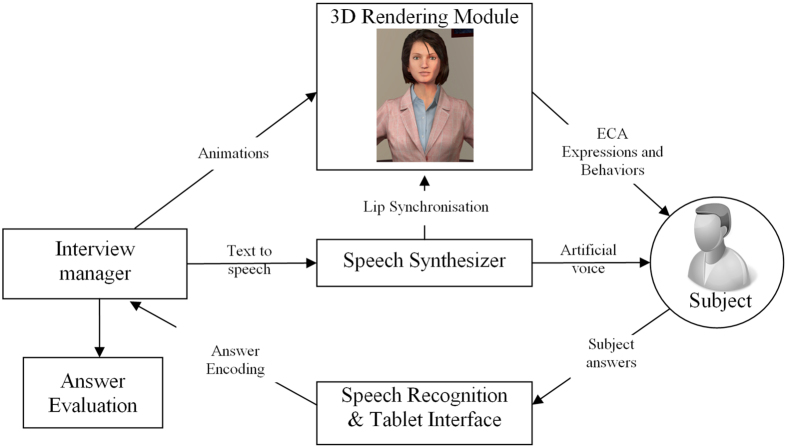
Architecture of the Embodied Conversational Agent used to self-administer interactive face-to-face clinical interviews based on Major Depressive Disorder DSM-5 criteria. The Embodied Conversational Agent was created on Unity https://unity3d.com/.

The software-driven virtual human was adapted from previously developed software designed to self-conduct interactive face-to-face clinical interviews^5^. The software is based on four modules:^5^ i) an interview manager that coordinates the whole interview and manages the other modules, ii) a 3D video module that displays the virtual human and plays animations, iii) a speech synthesizer that creates the speech of the virtual human, and iv) a speech recognizer that recognizes the responses of the patients. The software runs on a computer (Windows 8 - i7 3770@3.4 GHz − 8 GB - NVidia 670 GTX) connected to a 40-inch vertical display and to a Microsoft Kinect sensor. Figure 1 shows the overall design and interactive mode of the software-driven virtual human. The software-driven virtual human was judged as stable and robust.

The content of the interview is a sequence of questions based on DSM-5 criteria for MDD^3^. Fluency of the questions was optimized with iterative processes. The self-conducting interactive face-to-face clinical interview of the ECA was pre-tested on 19 patients before the study. This pre-test ensured that patients understood the task and the meaning of each question in the interview and that the interactions with the interface were dynamic.

### Statistics

Quantitative variables were expressed as Mean ± Standard Deviation (SD) and qualitative variables were expressed as relative frequency. Data analysis was performed using SPSS software (Version 18 for Mac, PASW Statistics) and MedCalc software (Version 14.8 for Windows). For all the tests, the accepted significance level was 5%.

Receiver Operating Characteristic (ROC) analysis was performed to test the validity of the diagnosis of MDD performed by the ECA in comparison with the clinical interview conducted by the psychiatrist. In statistics, a ROC curve is a graphical plot that illustrates the performance of a binary classifier system^23^,^24^. A ROC curve is a reference method for diagnostic test evaluation used to compare the diagnostic performance of two diagnostic tests (psychiatrist clinical interview versus ECA clinical interview in the present study)^25^. ROC curves make it possible to complete the area under the ROC curve (AUC), sensitivity/specificity and positive/negative predictive report. These parameters are a measure of how well a parameter can distinguish between two diagnostic groups (diseased/normal). Sensitivity is the probability that a test result will be positive when the disorder is present (true positive rate, expressed as a percentage). Specificity is the probability that a test result will be negative when the disorder is not present (true negative rate, expressed as a percentage). Positive predictive value is the probability that the disorder is present when the test is positive (expressed as a percentage). Negative predictive value is the probability that the disorder is not present when the test is negative (expressed as a percentage). Results were also stratified on severity of depressive symptoms according to the BDI-II.

## Results

### Participants

**Figure 2:**
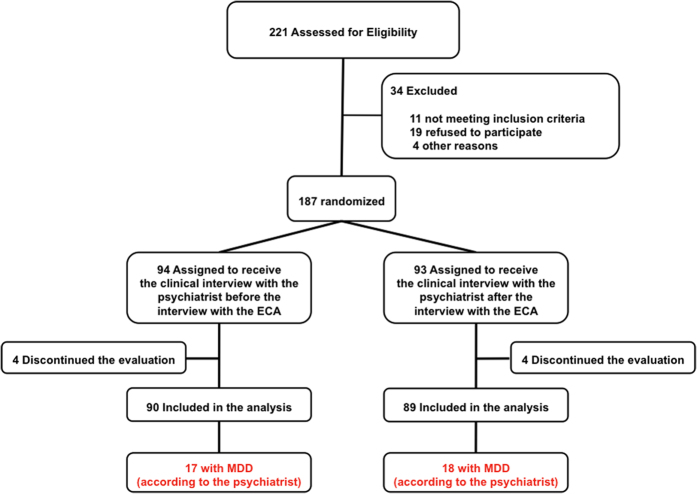
Flow chart that describes the patient selection and attribution process.

Out of 221 consecutive outpatients seen in consultation, 179 were available for data analysis, 90 received the clinical interview with the psychiatrist before the interview with the ECA and 89 the clinical interview with the psychiatrist after the interview with the ECA (Fig. 2). The mean age was 46.5 ± 12.9 years, 57.5% were females, mean educational level was 13.3 ± 3.0 years, and mean BDI-II score was 10.7 ± 9.3. Thirty-five patients (19.6%) were diagnosed with MDD by the psychiatrist.

In the group that received the clinical interview with the psychiatrist before the interview with the ECA, the mean age was 45.7 ± 13.6 years, 55.6% were females, mean educational level was 13.6 ± 3.2 years, and mean BDI-II score was 10.5 ± 9.6. In the group that received the clinical interview with the psychiatrist after the interview with the ECA, the mean age was 47.3 ± 12.1 years, 59.6% were females, mean educational level was 13.1 ± 2.9 years, and mean BDI-II score was 10.8 ± 9.0. No significant differences were found between the two groups (p > 0.05).

We tested the reliability and the validity of the BDI-II on our sample. Cronbach’s alpha coefficient was 0.89, which is an indicator of good reliability of this psychometric measure. The mean BDI-II score was higher in patients with MDD (22.3 ± 6.9) than in those without MDD (7.8 ± 7.3, p < 0.0001), which is an indicator of good validity.

### Global performance of ECA diagnosis of MDD in comparison with diagnosis

The ECA correctly encoded each response of the patient. The decisional algorithm tree implemented in the software-driven virtual human allowed a decision about the presence or absence of MDD to be calculated for each patient. Among the 35 patients who met the criteria of MDD according to the psychiatrist, the ECA correctly identified 17 MDD (true positives), while in 18 patients (false negatives) the ECA did not indicate MDD.

**Figure 3:**
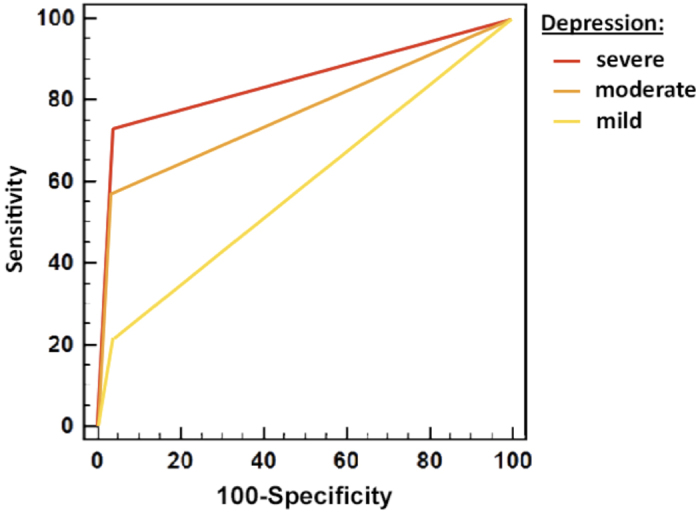
Receiver operating characteristics curve of ECA diagnosis of MDD stratified on severity of depressive symptoms (BDI-II).

The statistical ROC analysis of the ECA diagnosis of MDD showed an AUC of 0.71 (95% CI 0.59–0.81), (p < 0.001). The ROC analysis is shown in Fig. 3. The ECA had an overall sensitivity of 49% [31; 66], a specificity of 93% [88; 97], a positive predictive value (PPV) of 63% [42; 81], and a negative predictive value (NPV) of 88% [82; 93] Table 1.

### Stratified performance of ECA diagnosis of MDD based on severity of depressive symptoms

Among the 35 patients diagnosed with MDD by the psychiatrist, 14 (40%) patients exhibited mild, 12 (34.3%) moderate, and 9 (25.7%) severe depressive symptoms. Sensitivity increased across the severity level of depressive symptoms and reached 73% for patients with severe depressive symptoms (BDI-II ≥ 29), while specificity remained above 95% for all three severity levels. Sensitivity, specificity, positive/negative predictive values and true positives, false-negative and false-positive numbers stratified on severity of depressive symptoms according to the BDI-II are shown in Table 2.

### Acceptability of the ECA clinical interview

None of the patients reported any difficulties in understanding the sequence of questions from the ECA. None discontinued the clinical interview with the ECA. Eight patients discontinued the evaluation after the two clinical interviews (interviews with the psychiatrist and the ECA) mainly because of the duration of the global evaluation. The acceptability score was high (25.4 ± 4.6 on a scale of 0–30, 73% of the patients having a score above 24).

We tested the reliability and the validity of the AES in our sample. Cronbach’s alpha coefficient was 0.70, which is an indicator of good reliability of this psychometric measure. The correlation between AES and BDI-II scores was not significant (r = −0.12, p = 0.11) and the mean AES score was similar between women (25.12 ± 4.07) and men (25.53 ± 3.57, p = 0.48). Theses results suggest good validity of the AES, i.e the score was not related to the severity of depressive symptoms or to the sex of our subjects.

## Discussion

To our knowledge, this is the first study to evaluate the performance of an ECA to perform a diagnosis of major depressive disorders (MDD) using the DSM 5 criteria during a face-to-face clinical interview. Interestingly, about 20% of our patients seen in the sleep clinic reported MDD. This figure is consistent with previous studies and confirms the high prevalence of depression in the general population^17^.

Patients found the face-to-face interview with the ECA very acceptable according to the AES scale^19^,^22^. The good level of acceptability suggests that the ECA can communicate empathy^13^, elicit patient trust, reduce the feeling of being judged by a human and reduce emotional barriers to disclose an affective state. This is in line with previous studies that found that patients readily accepted new technological tools in medicine^26^. This very encouraging result should prompt more research into extending the use of software-driven virtual humans to other chronic diseases, keeping in mind that clinicians are generally more reticent than patients to use such tools^26^.

Such ECAs offer the advantage of being potentially in interaction with decision/information systems to develop new medical approaches for managing major chronic diseases and optimizing health care systems^27^. Such solutions could be very useful to diagnose chronic diseases early and follow them up, which is very costly and time-consuming for healthcare providers. Moreover, the shortage of physicians combined with an increase in the number of patients due to the ageing of the population highlights the need for new technologies to support physicians in their daily practice^28^. Even if the trend towards the development of electronic health (E-health) solutions is obvious, major challenges remain in their acceptance by patients^19^. The quality of human-machine interactions offered by ECAs is of paramount importance to promote their use. However, if ECA becomes the reference in the diagnosis and potential follow-up of patients, it will raise questions on the safety of information collected by the agents so a potential reticence could be observed in the medical community regarding the medical ethics related to these new tools. On the other hand, these systems could be integrated into decision support systems to optimize the follow up of chronic diseases and early interventions to prevent relapses. These new E-solutions now form part of the concept of precision medicine^29^ and are extremely suited to the field of psychiatry where there are no objective paraclinical measures of the symptoms of the patients, unlike in cardiology or neurology (i.e. EKG or CT scan).

The validity of the MDD diagnosis performed by the ECA in comparison with the gold standard diagnosis of the psychiatrist was satisfactory. Its specificity was also good irrespective of the severity of the depressive symptoms, so the ECA efficiently identified patients without MDD. Sensitivity increased significantly in patients with severe depressive symptoms, showing that the ECA can efficiently diagnose patients with severe MDD. However, its sensitivity was lower in patients with mildly or moderately depressive symptoms so work is required to refine its diagnostic accuracy. Improvements in the ECA’s semiologic non-verbal recognition skills (e.g. the ability to recognize facial expression or language prosody) and combining these with the expertise of affective computing research^13^ could lead to great progress in the field of psychiatry.

While face-to-face interviews are the gold standard to diagnose mental disorders, several problems limit the clinician’s capacity to conduct them efficiently. Firstly, only a limited time is available with each patient and the latter may feel intimidated about disclosing his/her affective state. Secondly, although such interviews are structured^30^, clinicians do not always follow the procedure in accordance with the recommended guidelines, so significant variations may occur. The present proof of concept study shows that ECAs are a promising tool for conducting replicable, standardized and well-accepted clinical interviews in medical settings. Their use could thus save time in the clinician’s busy daily schedule and increase the fidelity between different face-to-face interviews. These findings open up future perspectives for implementing ECAs in order to increase the satisfaction of patients with regard to the health-care system. Moreover, they could be incorporated into decision/information systems applied to medicine in order to increase the quality of care in chronic diseases.

## Additional Information

**How to cite this article**: Philip, P. *et al*. Virtual human as a new diagnostic tool, a proof of concept study in the field of major depressive disorders. *Sci. Rep.* **7**, 42656; doi: 10.1038/srep42656 (2017).

**Publisher's note:** Springer Nature remains neutral with regard to jurisdictional claims in published maps and institutional affiliations.

## Supplementary Material

Supplementary Information

## Figures and Tables

**Table 1:** Area Under the Curve, sensitivity, specificity, positive and negative predictive values with 95 percent confidence intervals [CI] of ECA diagnostic performance for total sample (psychiatrists’ diagnosis as standard reference).

ECA psychometric properties for identification of:	AUC	P value	Sensitivity	Specificity	Positive Predictive Value	Negative Predictive Value	True positives	False negatives	False positives
All patients	0.71 [0.59–0.81]	<0.001	49 [31–66]	93 [88–97]	63 [42–81]	88 [82–93]	17	18	10

Abbreviations: ECA = Embodied Conversational Agent; BDI = Beck Depression Inventory.

**Table 2:** Area Under the Curve, sensitivity and specificity values with 95 percent confidence intervals [CI] of ECA diagnostic performance, stratified on severity of depressive symptoms according to the BDI-II function of severity (mild, moderate and severe).

ECA psychometric properties for identification of:	AUC	P value	Sensitivity	Specificity
Mildly depressed patients (14≤BDI≤19)	0.58 [0.41–0.76]	0.28	21 [5–51]	96 [91–99]
Moderately depressed patients (20≤BDI≤28)	0.77 [0.61–0.93]	0.01	57 [29–82]	97 [92–99]
Severely depressed patients (29≤BDI≤63)	0.84 [0.68–1]	<0.001	73 [39–94]	96 [91–99]

Abbreviations: ECA = Embodied Conversational Agent.

BDI = Beck Depression Inventory.

## References

[b1] Shea S. Psychiatric Interviewing: the Art of Understanding. (Saunders Philadelphia, 1998).

[b2] American Psychiatric Association. Practice Guidelines for the Psychiatric Evaluation of Adults. Third Edition. (American Psychiatric Association, Arlington, 2015). 10.1176/appi.ajp.2015.1720501 26234607

[b3] American Psychiatric Association. Diagnostic and Statistical Manual of Mental Disorder. 5th ed. Text Revision (DSM-5). (American Psychiatric Association, Washington, DC, 2013).

[b4] Beck A. T., Steer R. A., Ball R. & Ranieri W. Comparison of Beck Depression Inventories -IA and -II in psychiatric outpatients. J Pers Assess 67(3), 588–597 (1996). 8991972 10.1207/s15327752jpa6703_13

[b5] Philip P., Bioulac S., Sauteraud A., Chaufton C. & Olive J. Could a Virtual Human be used to Explore Excessive Daytime Sleepiness in Patients? Presence: teleoperators and virtual environments 23(4), 369–376 (2014).

[b6] Bickmore T., Pfeifer L. & Paasche-Orlow M. Using computer agents to explain medical documents to patients with low health literacy. Patient Education and Counseling 315–20(75), 3 (2009). 10.1016/j.pec.2009.02.007 PMC2692364 19297116

[b7] Gorrindo T. & Groves J. E. Computer simulation and virtual reality in the diagnosis and treatment of psychiatric disorders. Acad Psychiatry 33(5), 413–417 (2009). 19828861 10.1176/appi.ap.33.5.413

[b8] Marks I., Cavanagh K. & Gega L. Hands-On Help: Computer-Aided Psychotherapy. (Psychology Press, New-York, 2007).

[b9] Glanz K., Rizzo A. & Graap K. Virtual reality for psychotherapy: Current reality and future possibilities. Psychotherapy: Theory, Research, Practice, Training 40(1–2), 55–67 (2003).

[b10] Proudfoot J. . Computerized, interactive, multimedia cognitive-behavioural program for anxiety and depression in general practice. Psychol Med 33(2), 217–227 (2003). 12622301 10.1017/s0033291702007225

[b11] Grime P. R. Computerized cognitive behavioural therapy at work: a randomized controlled trial in employees with recent stress-related absenteeism. Occup Med (Lond) 54(5), 353–359 (2004). 15289593 10.1093/occmed/kqh077

[b12] Cassell J., Sullivan J., Prevost S. & Churchill E. Embodied Conversational Agents. (MIT Press, Cambridge, 2000).

[b13] Cassell J. . Animated conversation: Rule-Based Generation of Facial Expression, Gesture & Spoken Intonation for Multiple Conversational Agents. Paper presented at SIGGRAPH ‘94 Proceedings of the 21st annual conference on Computer graphics and interactive techniques, Orlando, FL, USA. Place of publication: ACM New York, NY, USA 413–420. (1994 May).

[b14] Reeves B. & Nass C. The media equation: how people treat computers, television, and new media like real people and places. (Cambridge University Press, Cambridge, 1996).

[b15] Rizzo A. . Automatic Behavior Analysis During a Clinical Interview with a Virtual Human. Stud Health Technol Inform 220, 316–322 (2016). 27046598

[b16] Rizzo A. . SimCoach: an intelligent virtual human system for providing healthcare information and support. International Journal on Disability and Human Development 10(4), 277–281 (2011). 21335847

[b17] Bromet E. . Cross-national epidemiology of DSM-IV major depressive episode. BMC Med 9, 90 (2011). 21791035 10.1186/1741-7015-9-90 PMC3163615

[b18] Rodgers A. . Distribution of major health risks: findings from the Global Burden of Disease study. PLoS Med 1(1), e27 (2004). 15526049 10.1371/journal.pmed.0010027 PMC523844

[b19] Tariman J. D., Berry D. L., Halpenny B., Wolpin S. & Schepp K. Validation and testing of the Acceptability E-scale for web-based patient-reported outcomes in cancer care. Appl Nurs Res 24(1), 53–58 (2011). 20974066 10.1016/j.apnr.2009.04.003 PMC3030937

[b20] Bouvard M. Protocoles et échelles d'évaluation en psychiatrie et psychologie. (Elsevier Masson, Paris, 2010).

[b21] Bourque P. & Beaudette D. Psychometric study of the Beck Depression Inventory on a sample of French-speaking university students. Canadian Journal of Behavioural Science 14(3), 211–218 (1982).

[b22] Micoulaud-Franchi J. A. . Validation of the French version of the Acceptability E-scale (AES) for mental E-health systems. Psychiatry Res 237, 196–200 (2016). 26809367 10.1016/j.psychres.2016.01.043

[b23] Metz C. E. Basic principles of ROC analysis. Semin Nucl Med 8(4), 283–298 (1978). 112681 10.1016/s0001-2998(78)80014-2

[b24] Zweig M. H. & Campbell G. Receiver-operating characteristic (ROC) plots: a fundamental evaluation tool in clinical medicine. Clin Chem 39(4), 561–577 (1993). 8472349

[b25] Griner P. F., Mayewski R. J., Mushlin A. I. & Greenland P. Selection and interpretation of diagnostic tests and procedures. Principles and applications. Ann Intern Med 94(4 Pt 2), 557–592 (1981). 6452080

[b26] Waller R. & Gilbody S. Barriers to the uptake of computerized cognitive behavioural therapy: a systematic review of the quantitative and qualitative evidence. Psychol Med 39(5), 705–712 (2009). 18812006 10.1017/S0033291708004224

[b27] Chouvarda I. G., Goulis D. G., Lambrinoudaki I. & Maglaveras N. Connected health and integrated care: Toward new models for chronic disease management. Maturitas 82(1), 22–27 (2015). 25891502 10.1016/j.maturitas.2015.03.015

[b28] Caulfield B. M. & Donnelly S. C. What is Connected Health and why will it change your practice? QJM 106(8), 703–707 (2013). 23676416 10.1093/qjmed/hct114

[b29] Ashley E. A. The precision medicine initiative: a new national effort. JAMA 313(21), 2119–2120 (2015). 25928209 10.1001/jama.2015.3595

[b30] Sheehan D. V. . The Mini-International Neuropsychiatric Interview (M.I.N.I.): the development and validation of a structured diagnostic psychiatric interview for DSM-IV and ICD-10. J Clin Psychiatry 59 Suppl 20, 22-33;quiz 34–57 (1998). 9881538

