# Supplementary material for: Virtual human as a new diagnostic tool, a proof of concept study in the field of major depressive disorders
Source: Sci Rep. 2017 Feb 16;7:42656. doi: 10.1038/srep42656 (PMC5311989; doi:10.1038/srep42656)
Supplement: Supplementary Information [file srep42656-s1.doc]

**SUPPLEMENTARY MATERIALS**

***PROTOCOL & STATISTICAL ANALYSIS PLAN***

**TITLE**

**Virtual human as a new diagnostic tool, a proof of concept study in the field of major depressive disorders**

**AUTHORS**

Pierre PHILIP ^a,b,c *^, Jean-Arthur MICOULAUD-FRANCHI ^a,b,c^, Patricia SAGASPE ^a,b,c^, Etienne DE SEVIN ^b,c^, Jérôme OLIVE ^b,c^, Stéphanie BIOULAC ^b,c,d^, Alain SAUTERAUD ^b,c^

a - Clinique du Sommeil, Service d'Explorations Fonctionnelles du Système Nerveux, CHU de Bordeaux, Place Amélie Raba-Léon, 33076 Bordeaux, France

b – Univ. Bordeaux, SANPSY, USR 3413, F-33000 Bordeaux, France

c - CNRS, SANPSY, USR 3413, F-33000 Bordeaux, France

d - Pôle Universitaire Psychiatrie Enfants et Adolescents, Centre Hospitalier Charles Perrens, 121, rue de la Béchade, 33076 Bordeaux, France

*** Corresponding author:**

Pierre PHILIP, Clinique du Sommeil, Service d'Explorations Fonctionnelles du Système Nerveux, CHU de Bordeaux, Place Amélie Raba-Léon, 33076 Bordeaux, France.

E-mail address: pr.philip@free.fr

**TABLE OF CONTENTS**

**PROTOCOL**

[1. DEVELOPPMENT OF THE VIRTUAL AGENT 3](#__RefHeading___Toc436741276)

[1.1. ECA software 3](#__RefHeading___Toc436741277)

[1.2. Scrip implemented in the ECA software 4](#__RefHeading___Toc436741278)

[1.3. Pre-test 12](#__RefHeading___Toc436741279)

[2. CONDUCT OF THE CLINICAL TRIAL 13](#__RefHeading___Toc436741280)

[2.1. Clinical trial number 13](#__RefHeading___Toc436741281)

[2.2. Inclusion / Exclusion criteria 13](#__RefHeading___Toc436741282)

[2.3. Experimental conduct 13](#__RefHeading___Toc436741283)

[3. REFERENCES 14](#__RefHeading___Toc436741284)

**STATISTICAL ANALYSIS PLAN**

[1. DATA SOURCE 15](#__RefHeading___Toc436741285)

[1.1. Main variables 15](#__RefHeading___Toc436741286)

[1.2. Secondary variables 15](#__RefHeading___Toc436741287)

[2. ANALYSIS OBJECTIVES 15](#__RefHeading___Toc436741288)

[2.1. Main objective 15](#__RefHeading___Toc436741289)

[2.2. Secondary objective 16](#__RefHeading___Toc436741290)

[3. ANALYSIS SETS/SUB-GROUPS 16](#__RefHeading___Toc436741291)

[3.1. Entire group analyses 16](#__RefHeading___Toc436741292)

[3.2. Sub-group analyses 16](#__RefHeading___Toc436741293)

[4. RANDOMIZATION 17](#__RefHeading___Toc436741294)

[5. HANDLING OF MISSING DATA 17](#__RefHeading___Toc436741295)

[6. FLOW CHART 17](#__RefHeading___Toc436741296)

[7. STATISTICAL PROCEDURE 17](#__RefHeading___Toc436741297)

[7.1. Population description 17](#__RefHeading___Toc436741298)

[7.2. Psychometric properties of ECA 17](#__RefHeading___Toc436741299)

[7.2.1. Sensitivity, Specificity, Predictive values and Likelihood ratios 18](#__RefHeading___Toc436741300)

[7.2.2. Kappa values 18](#__RefHeading___Toc436741301)

[7.3. Acceptability 18](#__RefHeading___Toc436741302)

[8. REFERENCES 18](#__RefHeading___Toc436741303)

**PROTOCOL**

# DEVELOPPMENT OF THE VIRTUAL AGENT

The aim of this protocol is to develop a virtual agent (also called Embodied Conversational Agent, ECA) that is able to diagnose Major Depressive Episode according to the Diagnostic and Statistical Manual of Mental Disorders, Fifth Edition (DSM-V; American Psychiatric Association, 2013) depression criteria.

## ECA software

The ECA system used in the present study is based on four software modules that we already used in a previous study:

Philip P, et al. *Could a Virtual Human be used to Explore Excessive Daytime Sleepiness in Patients?* **Presence: teleoperators and virtual environments.** 2014;23(4):369-376.

The first and main module is defined as the interview manager. It conducts the whole interview (questions, expected answers, scripted gestures and scripted emotions) and manages the other modules. Instead of scripted behaviors, this module generates ECA behaviors based on predefined or random rules. All interviews are stored in XML files.

The second module is a 3D rendering module. Its role is to display our ECA and play animations on command. It was created with Unity3D (Unity-Technologies, 2014), a 3D gaming engine, and it uses 81-bone 3D characters from Rocketbox Libraries (RocketBox-Libraries, 2014). The characters can be animated in terms of gestures, facial expressions and visemes (facial expressions corresponding to enunciation of phonemes).

The third module operates the tablet interface and the speech recognizer. We use the speech recognition module from Microsoft Kinect SDK (Microsoft, 2014). The tablet interface is designed to assure the continuity of the system if the speech recognition fails by clicking the answer on the tablet. The interview manager feeds this module with dictionaries containing expected answers from participants. Answers of participants after analysis are transmitted to the interview manager.

The fourth and last module is a speech synthesizer. It creates ECA speech sent by the interview manager and, for each enunciated phoneme, sends the corresponding viseme command to the 3D rendering module.

These four modules are thread-independent (1). Modules communicate by TCP sockets that can be distributed to several computers.

The ECA software suite is installed on a standard gaming computer (Windows 8 - i7 3770@3.4GHz - 8 GB - NVidia 670 GTX) connected to a 40-inch display. As input device we use exclusively the Microsoft Kinect sensor for voice recognition and to monitor the user's head. The figure above describes the overall design and interactive mode of the ECA.

**Figure 1: ECA architecture and interactions**


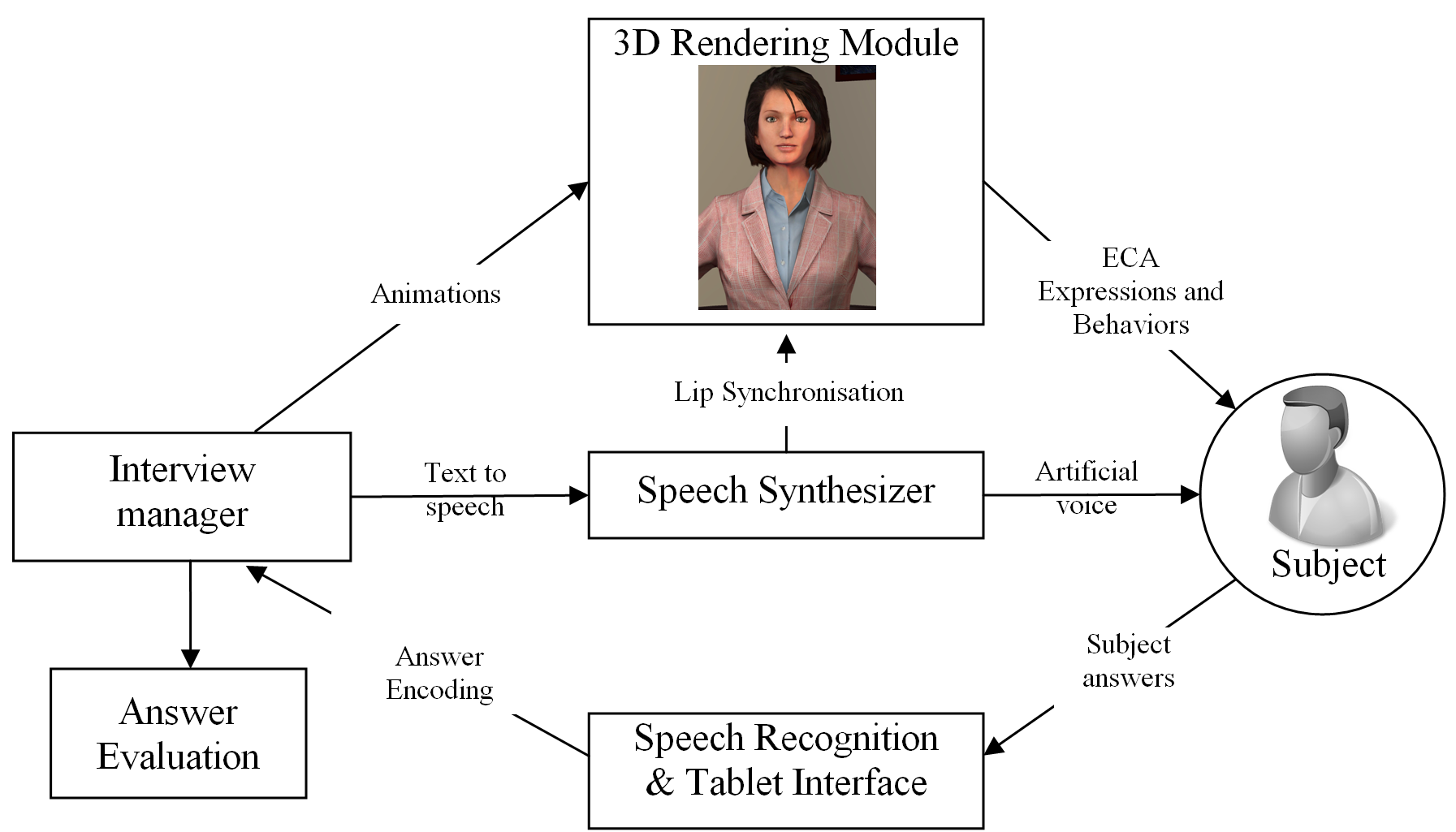


## Script implemented in the ECA software

The whole interview (questions, expected answers, scripted gestures and scripted emotions) is based on the Diagnostic and Statistical Manual of Mental Disorders, Fifth Edition (DSM-V; American Psychiatric Association, 2013) in order to investigate each depression criterion (2). Fluency of the question is optimized with iterative processes.

**Table 1: The entire French script implemented in the ECA software based on the Diagnostic and Statistical Manual of Mental Disorders, Fifth Edition**

| **Script implemented in the ECA software** | | | | | | |
| --- | --- | --- | --- | --- | --- | --- |
| Critère | ID | Phrases/Test (Sous-titre) | Diff dans Julia | Réponses | Renforcement/Action | Goto |
| Intro | _ | Bonjour! |  | _ | _ | _suivant |
|  | _ | Cette situation qui consiste pour vous de répondre à un écran peut vous paraître inhabituelle. | Cette situation qui consiste pour vous de parler à un écran peut vous paraitre inhabituelle. Pour moi aussi, c’est assez inhabituel d’être une psychiatre virtuelle. | _ | _ | _suivant |
|  | _ | Mais je vous assure que je vais prendre en compte attentivement votre état. Je serai très à l'écoute de vos réponses. |  |  | _ | _suivant |
|  | installé | Etes-vous bien installé ? |  | oui |  | _suivant |
|  |  |  |  | non | Très bien, je vous laisse un peu de temps. | installé |
|  | intro1 | Mon but aujourd'hui, avec vous, est d’examiner les moments difficiles de votre vie actuelle. |  | _ | _ | _suivant |
|  | intro2alt | Nous voulons savoir si vous souffrez en ce moment  de dépression, ou bien s'il s'agit seulement d'une mauvaise passe, comme chacun d’entre nous peut en vivre. |  | _ | _ | _suivant |
| D | D1 | Mon but aujourd'hui, avec vous, est d’examiner les moments difficiles de votre vie actuelle. Nous voulons savoir si vous souffrez en ce moment  de dépression, ou bien, s'il s'agit seulement d'une mauvaise passe, comme chacun d’entre nous peut en vivre. êtes-vous d'accord. |  | oui | _ | _suivant |
|  |  |  |  | non | Très bien, ce n'est pas grave je comprend que cela soit dur pour vous. Peut-être une prochaine fois. | _fin |
|  | _ | Très bien. Nous allons donc examiner plusieurs aspects de votre vie actuelle. Mais pour cela, je vais vous demander de vous concentrer sur vos deux dernières semaines |  |  | _ |  |
|  | D2 | Nous allons donc parler de ce qu’il s’est passé, de ce que vous avez ressenti ou de ce que vous avez fait au cours des 15 derniers jours.Cela vous semble-t-il possible. |  | oui | _ | D3 |
|  |  |  |  | non | _ | _suivant (D2b) |
|  | D2b | Je sais, c'est parfois difficile. On va cependant essayer, vous le voulez bien. |  | oui | _ | D3 |
|  |  |  |  | non | Je comprends, on pourra essayer plus tard, à un autre moment. D’ici là prenez soin de vous, et n’hésitez pas à me recontacter. | _fin |
|  | D3 | Mes questions attendent de votre part une réponse par oui ou par non. Mais c'est souvent difficile de choisir entre les deux; donc quand vous pensez, plutôt oui, répondez oui, et si vous pensez plutôt non répondez non. Suivez votre intuition, répondez naturellement.\|@_silence_300\|. Si vous n'avez pas compris une question, vous pourrez me demander de la répéter sur la tablette.\|@_silence_300\| Peut-on commencer. |  | oui | _ | _suivant |
|  |  |  |  | non | _ | _suivant |
| A1 | A1EtataTalk | Bien ! |  | _ | _ | _suivant |
|  | A1Etata | Je vous rappelle qu’il s’agit d’examiner votre état au cours des 15 derniers jours. Actuellement, avez-vous l'impression de vous sentir triste ou cafardeux au cours de ces 15 derniers jours ? |  | oui |  | _suivant |
|  |  |  |  | non | Bien | _suivant |
|  | A1Etatb | Vous sentez-vous déprimé ou désespéré en ce moment ? |  | oui | _ | _suivant |
|  |  |  |  | non | Très bien. | _suivant |
|  | A1Etatc | Votre entourage vous a-t-il fait remarquer que vous étiez déprimé, désespéré, triste ou cafardeux, même si vous, vous n’en êtes pas tout à fait conscient ? |  | oui | _ | _suivant |
|  |  |  |  | non | _ | _suivant |
|  | A1Etatc1 | Est-ce que l’on vous trouve triste à l'heure actuelle ? Ou bien vous a-t-on dit que vous pleurez facilement en ce moment ? |  | oui | _ | _suivant |
|  |  |  |  | non | _ | _suivant |
|  | A1etateval1 | [A1Etata]==1 OR [A1Etatb]==1 OR [A1Etatc]==1 OR [A1Etatc1]==1 |  | VRAI | _ | A1Dureea |
|  |  |  |  | FAUX | _ | A2Etata |
|  | A1Dureea | Actuellement, avez-vous l'impression que ces sentiments tristes sont présents la plupart du temps dans la journée ? \n Avant que vous ne répondiez, je vous précise que la plupart du temps, cela veut dire au moins la moitié du temps.\n Donc, je répète : avez-vous l’impression que ces sentiments sont présents la plupart du temps ? |  | oui | _ | _suivant |
|  |  |  |  | non | _ | _suivant |
|  | A1Dureeb | Au cours des deux dernières semaines, avez-vous l'impression que ces sentiments de tristesse, de cafard ou de dépression sont présents presque tous les jours ? |  | oui | _ | _suivant |
|  |  |  |  | non | _ | A1Dureea |
|  | A1Dureec | Ce doit être difficile pour vous de ressentir cela. Ces symptômes sont-il un changement dans votre façon de fonctionner.\n Donc habituellement, vous êtes différent. vous êtes moins triste ? |  | oui | _ | _suivant |
|  |  |  |  | non | _ | _suivant |
| A2 | A2Etata | Je vais vous poser une question difficile. Avez-vous l’impression que ce qui vous intéressait jusque-là a perdu de l’intérêt pour vous, ou bien vous procure moins de plaisir qu’auparavant ? |  | oui | _ | _suivant |
|  |  |  |  | non | Bien. Poursuivons. | _suivant |
|  | A2Etatabis | Avez-vous le sentiment de n’avoir plus goût à rien ? |  | oui | _ | _suivant |
|  |  |  |  | non | _ | _suivant |
|  | A2Etatb | Votre entourage vous fait-il remarquer cette perte d’intérêt, de goût ou de plaisir ? |  | oui | _ | _suivant |
|  |  |  |  | non | Merci de cette précision. | _suivant |
|  | A2etat | [A2Etata]==1 OR [A2Etatb]==1 |  | VRAI | _ | _suivant |
|  |  |  |  | FAUX | _ | A2DureeEval |
|  | A2Dureea | Cette perte de plaisir ou d’intérêt concerne-t-elle la plupart des activités de la journée ? Dit autrement : ressentez-vous cette indifférence au plaisir l’essentiel de la journée ? |  | oui | _ | _suivant |
|  |  |  |  | non | _ | _suivant |
|  | A2Dureeb | Nous venons de parler de ce qu’il se passe au sein d’une journée. A présent, je vais vous demander de vous concentrer sur ce que vous avez ressenti au cours des deux dernières semaines.\| Diriez-vous que vous ne ressentez ni intérêt, ni plaisir, la plupart des jours, c'est-à-dire plus d’un jour sur deux ? |  | oui | _ | _suivant |
|  |  |  |  | non | _ | _suivant |
|  | A2DureeEval | [A2Dureea]==1 AND [A2Dureeb]==1 |  | VRAI | _ | _suivant |
|  |  |  |  | FAUX | _ | _suivant |
|  | A2Continue | [A1]==0 AND [A2]==0 |  | VRAI | _ | _suivant |
|  |  |  |  | FAUX | _ | ENDtalk |
| A3 | A30 | Changeons à présent de sujet.|@_silence_500|Parlons maintenant de votre poids et de votre alimentation. |  |  | _ | _suivant |
|  | A31 | Votre appétit a-t-il significativement changé en plus, ou en moins au cours des deux dernières semaines. |  | oui | _ | _suivant |
|  |  |  |  | non | _ | _suivant |
|  | A31s | Très bien. Continuons |  | _ | _ | _suivant |
|  | A32 | Avez vous pris ou perdu du poids sans avoir fait de régime |  | oui | _ | _suivant |
|  |  |  |  | non | _ | A40 |
|  | A32b | Combien de kilos avez- vous pris ou perdu en un mois |  | POIDS | _ | _suivant |
|  | A32c | Quel était votre poids habituel avant cette période. |  | POIDS | _ | _suivant |
|  | A32EVAL | [A32b] >= (5.0/100*[A32c]) -> A32 |  | VRAI | _ | _suivant |
|  |  |  |  | FAUX | _ | _suivant |
|  | A3weight | [A31]==1 OR [A32]==1 -> A3 |  | VRAI | _ | _suivant |
|  |  |  |  | FAUX | _ | _suivant |
| A4 | A40 | Examinons à présent votre sommeil. |  | _ | _ | _suivant |
|  | A41 | Avez-vous des problèmes de sommeil, pratiquement toutes les nuits depuis au moins deux semaines ? |  | oui | _ | _suivant |
|  |  |  |  | non | _ | A42 |
|  | A41a | Avez-vous des difficultés à vous endormir le soir ? |  | oui | _ | _suivant |
|  |  |  |  | non | _ | _suivant |
|  | A41b | Au cours de la nuit, avez-vous des réveils trop fréquents ou trop longs, par rapport à votre sommeil habituel ? |  | oui | _ | _suivant |
|  |  |  |  | non | _ | _suivant |
|  | A41c | Vous réveillez-vous trop tôt le matin, et après, vous ne vous rendormez pas ? |  | oui | _ | _suivant |
|  |  |  |  | non | _ | _suivant |
|  | A42 | Dormez-vous plus longtemps que d’habitude ? |  | oui | _ | _suivant |
|  |  |  |  | non | _ | _suivant |
|  | A43 | Vous arrive-t-il de vous endormir sans le vouloir dans la journée ? |  | oui | _ | _suivant |
|  |  |  |  | non | _ | _suivant |
|  | A4Eval | (([A41]==1 AND ([A41a]==1 OR [A41b]==1 OR [A41c]==1 ) ) OR [A42]==1) |  | VRAI | _ | _suivant |
|  |  |  |  | FAUX | _ | _suivant |
| A5 | A50 | Nous allons parler maintenant de la façon dont vous vous sentez en forme ou sans énergie. |  | _ | _ | _suivant |
|  | A51a | Avez-vous l’impression d’être ralenti, dans vos gestes et vos actions ? |  | oui | _ | _suivant |
|  |  |  |  | non | _ | _suivant |
|  | A51b | Avez-vous l’impression d’une lenteur anormale comme si vous manquiez de force ou d’endurance ? |  | oui | _ | _suivant |
|  |  |  |  | non | _ | _suivant |
|  | A51eval | [A51a]==0 AND [A51b]==0 |  | VRAI | _ | A52 |
|  |  |  |  | FAUX | _ | _suivant |
|  | A51' | Si l’on reste à analyser la période actuelle, c'est-à-dire les deux dernières semaines, avez-vous cette impression presque tous les jours ? |  | oui | _ | _suivant |
|  |  |  |  | non | _ | _suivant |
|  | A52 | Au contraire, vous sentez-vous anormalement agité ou incapable de rester en place ? |  | oui | _ | _suivant |
|  |  |  |  | non | _ | _suivant |
|  | A52'" | Si l’on continue d’analyser seulement la période actuelle, c'est-à-dire les deux dernières semaines, avez-vous cette impression presque tous les jours ? |  | oui | _ | _suivant |
|  |  |  |  | non | _ | _suivant |
|  | A5eval | (([A51]==1 AND [A51']==1) OR ([A52]==1 AND [A52']==1)) -> A5 |  | VRAI | _ | _suivant |
|  |  |  |  | FAUX | _ | _suivant |
| A6 | A61 | Vous sentez-vous presque tout le temps épuisé ou sans énergie ? |  | oui | _ | _suivant |
|  |  |  |  | non | A6->0 | A61'bis |
|  | A61' | Si l’on réfléchit seulement depuis ces 15 derniers jours, ressentez-vous cette fatigue ou ce manque d’énergie tous les jours ou presque ? |  | oui | A6->1 | _suivant |
|  |  |  |  | non | A6->0 | _suivant |
|  | A61'bis | Quand vous effectuez des actes de la vie quotidienne, comme conduire ou faire les courses, vous sentez-vous somnolent ? |  | oui | _ | _suivant |
|  |  |  |  | non | _ | A70 |
|  | A61'ter | Cette somnolence vous oblige-t-elle à vous arrêter dans ce que vous faites ? |  | oui | _ | _suivant |
|  |  |  |  | non | _ | _suivant |
| A7 | A70 | Nous allons maintenant parler de tout autre chose. Je voudrais vous poser quelques questions sur ce que vous vous dites intérieurement. |  | _ | _ | _suivant |
|  | A71' | Parfois, on peut se faire des reproches. On peut se reprocher, par exemple, d’être tombé malade. Mais la question que je vais vous poser concerne des reproches plus douloureux. Avez-vous l’impression que vous n’êtes pas intéressant ? |  | oui | _ | _suivant |
|  |  |  |  | non | _ | _suivant |
|  | A71'' | Vous dites-vous que vous avez perdu de votre valeur ou que vous êtes sans valeur ? |  | oui | _ | _suivant |
|  |  |  |  | non | _ | _suivant |
|  | A71eval | ([A71']==0 AND [A71'']==0) |  | VRAI | _ | A72 |
|  |  |  |  | FAUX | _ | _suivant |
|  | A71b | Avez-vous cette impression presque tous les jours ces deux dernières semaines ? |  | oui | _ | _suivant |
|  |  |  |  | non | _ | A7 |
|  | A72 | Parfois nous pouvons ressentir un léger sentiment de culpabilité qui peut nous traverser ou nous faire réfléchir. Mais ma question va plus loin : avez-vous l’impression d‘être coupable de quelque chose d’important ? |  | oui | _ | _suivant |
|  |  |  |  | non | _ | _suivant |
|  | A72' | Ce point est essentiel. Je me permets d’insister : vous sentez-vous coupable de quelque chose que vous vous reprochez ? |  | oui | _ | _suivant |
|  |  |  |  | non | _ | A7 |
|  | A72b | Avez-vous cette impression presque tous les jours, au cours de ces deux dernières semaines ? |  | oui | _ | _suivant |
|  |  |  |  | non | _ | _suivant |
|  | A7 | ([A71]==1 AND [A71b]==1) OR ([A72]==1 AND [A72b]==1) |  | VRAI | _ | _suivant |
|  |  |  |  | FAUX | _ | _suivant |
| A8 | A80 | Nous allons à présent parler de votre activité intellectuelle. |  |  | _ | _suivant |
|  | A81a | Avez-vous l’impression d’avoir du mal à réfléchir en ce moment. |  | oui | _ | _suivant |
|  |  |  |  | non | _ | _suivant |
|  | A81b | Avez-vous du mal à vous concentrer ? |  | oui | _ | _suivant |
|  |  |  |  | non | _ | _suivant |
|  | A81c | Eprouvez-vous des difficultés à prendre des décisions ? |  | oui | _ | _suivant |
|  |  |  |  | non | _ | _suivant |
|  | A81 | ([A81a]==0 AND [A81b]==0 AND [A81c]==0)? |  | VRAI | A81 ->0; A82 ->0 | A83 |
|  |  |  |  | FAUX | A81->1 | _suivant |
|  | A82 | Avez-vous cette impression la plupart des jours, c'est-à-dire presque tous les jours ? |  | oui | A83 ->0 | A8 |
|  |  |  |  | non | _ | _suivant |
|  | A83 | Est ce que votre entourage vous l’a fait remarquer ? |  | oui | _ | _suivant |
|  |  |  |  | non | _ | _suivant |
|  | A83talk | Merci, continuons |  | _ | _ | _suivant |
|  | A8 | ([A81]==1 AND [A82]==1 OR [A83]==1)? |  | VRAI | _ | _suivant |
|  |  |  |  | FAUX | _ | _suivant |
| A9 | A90 | Je dois maintenant parler avec vous d’un sujet qui peut être douloureux. C’est le problème des pensées très tristes. |  | _ | _ | _suivant |
|  | A91 | Actuellement avez-vous des idées noires, ou des idées de mort ? |  | oui | _ | _suivant |
|  |  |  |  | non | A92->0 | A93 |
|  | A92 | Je précise ma question. Je ne vous demande pas si vous avez peur de la mort. Ma question en fait est la suivante : pensez-vous qu’il vaudrait mieux que vous soyez mort ? |  | oui | _ | _suivant |
|  |  |  |  | non | _ | _suivant |
|  | A93 | Pensez-vous à vous faire du mal actuellement, même si cette pensée est peu fréquente ? |  | oui | Très bien A94->0 | A95 |
|  |  |  |  | non | _ | _suivant |
|  | A94Talk | Je comprends que c’est peut-être désagréable pour vous, mais il me faut explorer cette question délicate. |  | _ | _ | _suivant |
|  | A94 | Avez-vous eu ces deux dernières semaines de telles idées de mort ? |  | non | _ | _suivant |
|  |  |  |  | oui | _ | _suivant |
|  | A95 | Je sais qu’il est compliqué d’aborder une question pénible comme celle-ci, mais je dois vous poser la question : avez-vous tenté de vous faire du mal au cours de ces deux dernières semaines ? |  | non | _ | _suivant |
|  |  |  |  | oui | _ | _suivant |
|  | A96 | Et actuellement, avez-vous imaginé comment vous pourriez faire pour vous suicider ? |  | non | _ | _suivant |
|  |  |  |  | oui | _ | _suivant |
|  | A9eval | ([A91]==1 OR [A92]==1 OR [A93]==1 OR [A94]==1 OR [A95]==1 OR [A96]==1) |  | VRAI | _ | _suivant |
|  |  |  |  | FAUX | _ | _suivant |
| B | B0 | J'imagine que c’est peut-être une période douloureuse pour vous. Peut-être avez-vous déjà traversé de tels moments. Mais peut-être même que le fait seul d’en parler est difficile. |  | _ | _ | _suivant |
|  | B1 | Ma question concerne le retentissement dans votre vie, de l'ensemble de ces problèmes. Parfois, on a des périodes difficiles mais on continue à vivre, sans trop y penser. Mais chez vous, ces problèmes entraînent-ils une souffrance importante ? |  | non | Merci de votre réponse. | _suivant |
|  |  |  |  | oui | _ | _suivant |
|  | B2 | Ces signes, que nous avons décrits, vous gênent-ils dans votre travail ? |  | non | _ | _suivant |
|  |  |  |  | oui | _ | _suivant |
|  | B3 | Pensez-vous, qu’à cause de votre état actuel, vos relations avec votre famille, vos amis ou votre entourage sont perturbées ? |  | non | Bien. Passons à la question suivante. | _suivant |
|  |  |  |  | oui | _ | _suivant |
|  | B4 | Nous avons parlé du travail et de vos relations, mais à coté de cela, ressentez-vous une perturbation importante dans un loisir qui vous tient à cœur, comme un sport ou bien votre activité préférée ? |  | non | _ | _suivant |
|  |  |  |  | oui | _ | _suivant |
|  | Beval | ([B2]==1 OR [B3]==1 OR [B4]==1) ? |  | VRAI | B->1 | _suivant |
|  |  |  |  | FAUX | B->0 | _suivant |
| C | C0 | Nous avons bien avancé, il y a encore deux points importants qu’il faut aborder : votre santé et la prise de produits. |  | _ | _ | _suivant |
|  | C1Talk | Parlons d’abord de votre santé.\nMa question concerne votre santé en général. |  |  | _ | _suivant |
|  | C1 | Est-ce que vous souffrez actuellement d’une maladie ou d’un problème de santé quel qu’il soit, qui pourrait expliquer en partie vos problèmes psychologiques actuels ? |  | non | _ | C1'' |
|  |  |  |  | oui | _ | _suivant |
|  | C1' | Allons un peu plus loin. On peut se sentir déprimé en cas d’infection comme une hépatite virale, ou bien en cas de problèmes de thyroïde, d’hypertension ou d’opération chirurgicale récente.\| Il y a tant de maladies possibles... Est-ce que vous souffrez d’un autre problème de santé, actuellement, en dehors de la dépression naturellement ? |  | non | _ | _suivant |
|  |  |  |  | oui | _ | _suivant |
|  | C1'' | Avez-vous subi une opération chirurgicale dans ces 6 derniers mois ? |  | non | _ | _suivant |
|  |  |  |  | oui | _ | _suivant |
|  | C2Talk | Merci de cette précision. |  | _ | _ | _suivant |
|  | C2 | Ces six derniers mois, avez-vous pris des médicaments que vous ne preniez pas auparavant ? |  | non | _ | _suivant |
|  |  |  |  | oui | _ | _suivant |
|  | C3Talk | Je dois maintenant vous demander si vous prenez des produits qui pourraient influencer votre santé. \| Je vous rappelle que notre entretien est soumis au secret médical et que personne d’autre qu’un médecin ne peut avoir accès à vos réponses. |  | _ | _ | _suivant |
|  | C3 | Actuellement, est-ce que vous consommez des produits comme du haschich, de la cocaïne ou de l'ecstasy ou une autre drogue ? |  | non | _ | _suivant |
|  |  |  |  | oui | _ | _suivant |
|  | C4 | Parlons à présent de l’alcool. Est-ce que vous consommez, tous les jours ou presque, 3 verres de vin par jour ou plus ? |  | non | _ | _suivant |
|  |  |  |  | oui | _ | _suivant |
|  | C5 | Est-ce que vous buvez quotidiennement ou presque, de l’alcool comme de la bière, du whisky ou un apéritif ? |  | non | _ | _suivant |
|  |  |  |  | oui | _ | _suivant |
|  | CEval | ([C1]==1 OR [C1']==1 OR [C2]==1 OR [C3]==1 OR [C4]==1 OR [C5]==1) ? |  | VRAI | _ | _suivant |
|  |  |  |  | FAUX | _ | _suivant |
| P | P0 | Merci de vos réponses.| Nous allons bientôt pouvoir nous quitter.| Mais j’ai encore trois dernières questions à vous poser concernant des événements pénibles qui ont pu se produire. Ceux-ci pourraient expliquer votre état actuel. |  | _ | _ | _suivant |
|  | P1 | En premier, la question d’un éventuel deuil : avez-vous perdu un être cher récemment ? |  | non | P1'->0 | P2 |
|  |  |  |  | oui | _ | _suivant |
|  | P1' | Pensez-vous que ce deuil puisse expliquer votre état actuel ? |  | non | _ | _suivant |
|  |  |  |  | oui | _ | _suivant |
|  | P2 | Merci de m’avoir répondu.\| Je change un peu de sujet en parlant de votre situation financière. Avez-vous fait de très mauvaises affaires, même si vous n’êtes pas ruiné ? |  | non | P2'->0 | P3 |
|  |  |  |  | oui | _ | _suivant |
|  | P2' | Pensez-vous que ces difficultés financières ont entraîné votre état actuel ? |  | non | _ | _suivant |
|  |  |  |  | oui | _ | _suivant |
|  | P3 | En dehors d’un deuil ou d'importants problèmes d’argent, avez-vous subi un évènement gravissime, comme un accident sévère, une agression physique grave ou une catastrophe naturelle, par exemple un ravage par inondation ou par la foudre ? |  | non | P3'->0 | _suivant |
|  |  |  |  | oui | _ | _suivant |
|  | P3' | Pensez-vous que votre état actuel est la conséquence de cet événement ? |  | non | _ | _suivant |
|  |  |  |  | oui | _ | _suivant |
|  | Peval | ([P1]==1 OR [P1']==1 OR [P2]==1 OR [P2']==1 OR [P3]==1 OR [P3']==1) ? |  | VRAI | P->1 | _suivant |
|  |  |  |  | FAUX | P->0 | _suivant |
| END | ENDtalk | Voilà, nous avons fini. Je vous remercie et je vous félicite. C’est difficile de réfléchir sur soi, et d’examiner tout ce qui ne va pas en nous. |  | _ | _ | _suivant |
|  | END | J’ai été heureux de vous rencontrer. Vous pouvez maintenant poser la tablette.\n Je vous souhaite une bonne journée.\nÀ bientôt peut-être. | J’ai été heureuse de vous rencontrer.\|@_silence_500\| Vous pouvez maintenant poser la tablette.\|@_silence_500\| Je vous souaite une bonne journée. A bientôt peut-être. | _ | _ | _suivant |
| EVAL Dep | | ([A1]+[A2]+[A3]+[A4]+[A5]+[A6]+[A7]+[A8]+[A9] >=5) AND ([A1]==1 OR [A2]==1) AND [B]==1 )?1:([C]==1 OR [P]==1)?2:0 |  | 0 | non depressif | _end |
|  |  |  |  | 1 | depressif |  |
|  |  |  |  | 2 | On ne peut conclure car P et C |  |

## Pre-test

The interface will be tested on 19 patients who do not belong to the group of patients tested in the submitted study. The pre-test is designed to ensure that patients (a) understand the task well and in particular the meaning of each question in the interview; (b) easily answer with the interface; and (c) that data is recorded, correctly stored, and easy to export to a statistic software.

# CONDUCT OF THE CLINICAL TRIAL

## Clinical trial number

The study was approved by the local ethics committee and declared as a clinical trial (ClinicalTrials.gov identifier: NCT02544295).

## Inclusion / Exclusion criteria

Outpatients will be recruited at Bordeaux University Hospital from November 2014 to June 2015 in a consecutive sample design.

The inclusion criteria are:

- To be aged from 18 to 65 years,

- To be a French native speaker,

- To provide written informed consent.

The exclusion criteria are mental, visual or auditory deficits interfering with ECA interaction.

## Experimental conduct

Patient included in this study have to

- meet a psychiatrist who conducts a medical interview based on the Diagnostic and Statistical Manual of Mental Disorders, Fifth Edition (DSM-V; American Psychiatric Association, 2013) depression criteria in order to diagnose (yes, no) a major depressive episode,
- meet the ECA software, which we call Julia (a female ECA medical doctor), which conducts a medical interview based on the Diagnostic and Statistical Manual of Mental Disorders, Fifth Edition (DSM-V; American Psychiatric Association, 2013) depression criteria in order to diagnose (yes, no) a major depressive episode,
- complete the French version of the Tariman Acceptability E-scale (AES) (3).
- complete the BDI-II score (Beck Depression Inventory BDI II) to quantify depressive symptoms (4).

The clinical interviews with a psychiatrist and with the ECA (Julia) are performed in a randomized order. The AES and the BDI II scales are completed after the clinical interviews.

# REFERENCES

1. Philip P, Bioulac S, Sauteraud A, Chaufton C, Olive J. Could a Virtual Human be used to Explore Excessive Daytime Sleepiness in Patients? Presence: teleoperators and virtual environments. 2014;23(4):369-76.

2. American Psychiatric Association. Diagnostic and Statistical Manual of Mental Disorder, 5th ed, Text Revision (DSM-5). Washington, DC: American Psychiatric Association; 2013.

3. Tariman JD, Berry DL, Halpenny B, Wolpin S, Schepp K. Validation and testing of the Acceptability E-scale for web-based patient-reported outcomes in cancer care. Appl Nurs Res. 2011 Feb;24(1):53-8.

4. Beck AT, Steer RA, Ball R, Ranieri W. Comparison of Beck Depression Inventories -IA and -II in psychiatric outpatients. J Pers Assess. 1996 Dec;67(3):588-97.

**STATISTICAL ANALYSIS PLAN**

# DATA SOURCE

Two conditions allow each to obtain a depression diagnosis in a categorical judgment (yes/no) for the same patient: (1) Interview by an experienced psychiatrist and (2) Structured interview by an ECA.

At the end of the protocol, patients complete scales to evaluate depression and the acceptability of the ECA.

## Main variables

- Diagnosis (yes, no) of the psychiatrist (reference standard): A psychiatrist diagnoses patients suffering from a major depressive episode according to the Diagnostic and Statistical Manual of Mental Disorders, Fifth Edition (DSM-V; American Psychiatric Association, 2013) depression criteria.

- Diagnosis (yes, no) of the Embodied Conversational Agent (ECA): Based on a structured interview, a female ECA medical doctor diagnoses (yes, no) patients according to the Diagnostic and Statistical Manual of Mental Disorders, Fifth Edition (DSM-V; American Psychiatric Association, 2013) depression criteria.

## Secondary variables

- Acceptability score for the ECA: Evaluated with the French version of the Tariman Acceptability E-scale (AES) .

- BDI-II score: Patients complete a depression severity scale (Beck Depression Inventory BDI II) to quantify depressive symptoms.

# ANALYSIS OBJECTIVES

## Main objective

Our main objective is to test the validity of a virtual agent performing a DSM-5 based interview to discriminate patients with MDD from those without MDD.

Considering the psychiatrist’s diagnosis as the standard reference, analyses of the psychometrics properties of the ECA are computed:

- Probability of a positive test if the patient is depressed (Sensitivity)

- Probability of a negative test if the patient is non-depressed (Specificity)

- Proportion of positive results that are true positives (Positive predictive value)

- Proportion of negative results that are true negatives (Negative predictive value)

- Ratio of the proportion of depressed patients who have a positive test to the proportion of non-depressed patients who have also a positive test (Positive likelihood ratio)

- Ratio of the proportion of depressed patients who have a negative test to the proportion of non-depressed patients who also have a negative test (Negative likelihood ratio)

The degree of agreement between the psychiatrist and the ECA diagnoses is computed with Cohen’s kappa coefficient.

## Secondary objective

The secondary objective is to test the acceptability of a virtual agent performing a DSM-5 based interview to discriminate patients with MDD from those without MDD.

The T-test is computed to compare acceptability scores between depressed and non-depressed patients. A linear regression is computed to explore the association between acceptability scores and BDI scores.

# ANALYSIS SETS/SUB-GROUPS

## Entire group analyses

Population description is examined for the entire group.

ECA psychometrics properties are examined for the entire group.

The association between acceptability and severity of depression is examined for the entire group.

## Sub-group analyses

Population description is examined in two sub-groups (patients with MDD and those without MDD) according to the psychiatrist’s diagnosis.

Psychometric properties of the ECA are examined in three sub-groups according to the severity of the depressive symptoms (BDI-II).

Acceptability is examined in two sub-groups (patients with MDD and those without MDD) according to the diagnosis of the psychiatrist.

# RANDOMIZATION

Patient allocation to the diagnostic sequence will be randomized. The investigators will be kept ignorant of the sequence order and of the results of the previous assessment, if any.

# HANDLING OF MISSING DATA

Missing data are ignored if distinct from diagnosis of the psychiatrist, diagnosis of the ECA or from BDI scores in the statistical analysis.

If missing data concern the psychiatrist’s diagnosis, the diagnosis of the ECA or BDI scores, the subject is not considered for statistical analyses.

# FLOW CHART

A flow chart will describe the patient selection and attribution process.

# STATISTICAL PROCEDURE

The Alpha risk threshold is set at p = .05. Statistical analyses are performed using the SPSS statistical software package (PASW® Statistics 18).

## Population description

Quantitative variables are expressed as Mean ± Standard Deviation (SD) and qualitative variables are expressed as relative frequency.

## Psychometric properties of ECA

Patients are categorized according to the severity of their depressive symptoms assessed by the BDI-II total score as follows: 0 to 13 as non-depressed or minimally depressed, 14 to 19 as mildly depressed, 20 to 28 as moderately depressed, and 29 to 63 as severely depressed. These cut-offs are suggested by the revised manual of the BDI-II as guidelines for interpreting specific levels of the severity of depressive symptoms .

### Sensitivity, Specificity, Predictive values and Likelihood ratios

Considering the psychiatrist’s diagnosis as standard reference, predictive indices are calculated for the entire group of patients and according to the severity of depression as described above:

a) Sensitivity

b) Specificity

c) Positive predictive value

d) Negative predictive value

e) Positive likelihood ratio

f) Negative likelihood ratio

### Kappa values

Cohen’s kappa coefficient is calculated to evaluate the degree of agreement between the psychiatrist and the ECA diagnoses above that expected by chance.

## Acceptability

T-tests for independent means are used to compare acceptability scores between depressed and non-depressed patients. A linear regression adjusted for gender, age and educational level is performed to explore the association between acceptability scores and BDI scores.

# REFERENCES

1. Tariman JD, Berry DL, Halpenny B, Wolpin S, Schepp K. Validation and testing of the Acceptability E-scale for web-based patient-reported outcomes in cancer care. Appl Nurs Res. 2011 Feb;24(1):53-8.

2. Beck AT, Steer RA, Brown GK. Manual for the Beck Depression Inventory-II. San Antonio, TX: Psychological Corporation. 1996.

3. Landis JR, Koch GG. The measurement of observer agreement for categorical data. Biometrics. 1977 Mar;33(1):159-74.
